# *AtHMA4* Drives Natural Variation in Leaf Zn Concentration of *Arabidopsis thaliana*

**DOI:** 10.3389/fpls.2018.00270

**Published:** 2018-03-01

**Authors:** Zi-Ru Chen, Lu Kuang, Yi-Qun Gao, Ya-Ling Wang, David E. Salt, Dai-Yin Chao

**Affiliations:** ^1^National Key Laboratory of Plant Molecular Genetics, CAS Center for Excellence in Molecular Plant Sciences, Institute of Plant Physiology and Ecology, Shanghai Institutes for Biological Sciences, Chinese Academy of Sciences, Shanghai, China; ^2^University of Chinese Academy of Sciences, Beijing, China; ^3^School of Life Sciences, Jiangsu Normal University, Xuzhou, China; ^4^Division of Plant and Crop Sciences, School of Biosciences, University of Nottingham, Loughborough, United Kingdom

**Keywords:** *Arabidopsis thaliana*, BSA, cadmium, Heavy Metal ATPase 4, natural variation, QTL, zinc

## Abstract

Zinc (Zn) is an essential element for plant growth and development, and Zn derived from crop plants in the diet is also important for human health. Here, we report that genetic variation in *Heavy Metal-ATPase 4* (*HMA4*) controls natural variation in leaf Zn content. Investigation of the natural variation in leaf Zn content in a world-wide collection of 349 *Arabidopsis thaliana* wild collected accessions identified two accessions, Van-0 and Fab-2, which accumulate significantly lower Zn when compared with Col-0. Both quantitative trait loci (QTL) analysis and bulked segregant analysis (BSA) identified *HMA4* as a strong candidate accounting for this variation in leaf Zn concentration. Genetic complementation experiments confirmed this hypothesis. Sequence analysis revealed that a 1-bp deletion in the third exon of *HMA4* from Fab-2 is responsible for the lose of function of *HMA4* driving the low Zn observed in Fab-2. Unlike in Fab-2 polymorphisms in the promoter region were found to be responsible for the weak function of *HMA4* in Van-0. This is supported by both an expression analysis of *HMA4* in Van-0 and through a series of T-DNA insertion mutants which generate truncated *HMA4* promoters in the Col-0 background. In addition, we also observed that Fab-2, Van-0 and the *hma4-2* null mutant in the Col-0 background show enhanced resistance to a combination of high Zn and high Cd in the growth medium, raising the possibility that variation at *HMA4* may play a role in environmental adaptation.

## Introduction

Zinc (Zn) is an essential micronutrient for plants, as it plays important roles in many biological processes, including as a co-factor of various enzymes and transcription factors (Broadley et al., 2012). However, high concentrations of Zn are toxic to plants (Lin and Aarts, 2012). The Zn concentration in plant cells is therefore finely tuned. In addition, Zn is an essential micronutrient crucial for the health of livestock and humans, and plants are a major dietary source of Zn. Therefore, understanding the mechanism of Zn accumulation in plants is not only important for plant nutrition but is also of significance for human health and the health of livestock animals. Zn accumulation and utilization in plants are regulated by multiple processes, including Zn acquisition and uptake from the soil, radial transport across the root through the cortex and endodermis, long-distance transport from root to shoot, and distribution and redistribution among different tissues and organs. These processes involve various Zn transporters expressed in specific tissues and cell types. For example, the expression of a group of Zinc-regulated transporter/Iron-regulated transporter (ZRT/IRT)-like proteins (ZIP) are responsive to Zn deficiency, and function in the uptake of Zn from the rhizosphere and the distribution of Zn among different tissues and cells (Grotz et al., 1998). Two basic-region leucine-zipper (bZIP) type of transcription factors, bZIP19 and bZIP23 play key roles in regulating the expression of those *ZIP* genes in response to Zn status (Assuncao et al., 2010).

The P-type ATPase family functions in transport of various cations mainly across membranes and can be divided into different subfamilies according to sequence similarities (Axelsen and Palmgren, 1998). Among those subfamilies, type 1_B_ ATPases function in transport of heavy metals such Zn, Cd, Cu, Ag, Pb, and Co, and were so named Heavy Metal ATPases (HMA). There are eight *HMA* genes in the *A. thaliana* genome. Among them *HMA2, HMA3* and *HMA*4 are closely related in sequence. Genetic and molecular evidences have established that *HMA3* is involved in controlling vacuole sequestration of Zn and Cd, while *HMA4* and *HMA2* control root-to-shoot long distance transport of Zn and Cd, with *HMA4* playing the major role (Hussain et al., 2004; Hanikenne et al., 2008; Morel et al., 2009). GFP-tagging and promoter-reporter constructs revealed that HMA2 and HMA4 localize on the plasma membrane of the root pericycle (Hussain et al., 2004; Sinclair et al., 2007). HMA2 and HMA4 have been proposed to function in loading of Zn and Cd from the pericycle into the xylem for long-distance transport to the shoot, which is consistent with the efflux function of these proteins. This role is further supported by the cell-type specific localization of Zn in roots of wild type, *hma4* and the *hma2hma4* double mutant using the Zn-specific fluorophore Zinpyr-1 (Sinclair et al., 2007).

Interestingly, a previous study demonstrated that enhanced activity of *HMA4*, through both increased promoter activity and increased gene copy numbers, is necessary for the ability of *Arabidopsis halleri* to hyperaccumulate Zn and Cd (Hanikenne et al., 2008). Similarly, increased copy number of *HMA4* was also observed in the Zn/Cd hyperaccumulator *Noccaea caerulescens* (Craciun et al., 2012).

Natural variation within species has been shown to be a powerful tool for the investigation of both gene function and the role of such variation in local adaptation to specific environments (Todesco et al., 2010; Weigel, 2012; Li et al., 2015; Wang and Qin, 2017). Previous studies have shown that the genetic diversity of *A. thaliana* is extensive across its global range (Cao et al., 2011; Horton et al., 2012), and at least part of this genetic variation controls phenotypic variability and local adaptation to specific environments (Baxter et al., 2010; Fournier-Level et al., 2011; Hancock et al., 2011; Kronholm et al., 2012). Multiple genetic factors controlling natural variations in the concentrations of various mineral elements in leaves have been identified using a combination of forward genetics, reverse genetics, genome-wide association studies (GWAS) and mapping in bi-parental recombinant inbred populations (Rus et al., 2006; Tomatsu et al., 2007; Baxter et al., 2008; Chao et al., 2012, 2014a,b; Campbell et al., 2017) and some of this variations may be adaptive (Baxter et al., 2010; Poormohammad Kiani et al., 2012). In this study, we use a similar strategy to identify the genetic basis underlying natural variation in leaf Zn concentration.

## Materials and Methods

### Plant Materials and Growth Conditions

The 349 natural accessions used in this study were selected from 5810 worldwide *A. thaliana* accessions that were described previously (Baxter et al., 2010; Platt et al., 2010; Chao et al., 2012). The *HMA4* T-DNA insertion mutants [SALK_041334, FLAG_443D02, SALK_060112, FLAG_067a07, SALK_019060, SALK_066029, SALK_132258 and SALK_050924 *(hma4-2*)] were obtained from the Nottingham Arabidopsis Stock Center (NASC) or the Arabidopsis Biological Resource Center (ABRC). All of the plants used for leaf zinc analysis were grown in a controlled environment for 5 weeks as previously described (Lahner et al., 2003; Chao et al., 2012), and plants cultivated as previously described (Baxter et al., 2007, 2010; Chao et al., 2012; Danku et al., 2013). Plants used for studying the expression level of *HMA4* and phenotypes in different concentrations of Zn and Cd were grown in axenic conditions. Seeds were sterilized using 75% ethyl alcohol for 1min followed by 10% bleach for 10 min, and then washing at least 8 times with sterilized deionized water. The surface sterilized seeds were sown on 1x Hoagland media Ca(NO_3_)_2_: 2 mM, KNO_3_: 2 mM, NH_4_NO_3_: 0.5 mM, MgSO_4_: 0.5 mM, KH_2_PO_4_: 0.25 mM, KCl: 0.5 mM, Fe-EDTA: 40 μM, H_3_BO_3_: 2.5 μM, MnCl_2_: 2 μM, ZnSO_4_: 2 μM, CuSO_4_: 0.5 μM, CoCl: 0.15 μM, (NH_4_)_6_⋅Mo_7_O_24_: 0.0075 μM) containing 1% sucrose and solidified with (1.2%) agar. The sowing seeds were placed for 3 days at 4°C for stratification, and then maintained at 16 h light (120 μmol⋅m^-2^⋅s^-1^) and 8 h dark and 22°C. After 5-day plants were moved to new solidified Hoagland media with different Zn and Cd concentrations and allowed to grow in 16 h light (120 μmol⋅m^-2^⋅s^-1^) and 8 h dark and 22°C for a further 10 days.

### Elemental Analysis

The concentration of various elements in plant tissue was determined using an inductively coupled plasma mass spectrometer (ICP-MS), as described previously (Lahner et al., 2003; Danku et al., 2013). Briefly, one or two adult rosette leaves (2–4 mg dry weight) were collected from 5-week-old plants, followed by rinsing with ultrapure water (18.2 MΩ-cm MilliQ, Merck Millipore). Leaf samples were dried at 88°C in Pyrex tubes for 20 h. For each ICP-MS analysis sample set 7 samples from various genotypes were weighed, after cooling, and used as part of the weight normalization procedure (Lahner et al., 2003). All dried samples, together with blank controls, were digested with 0.7 mL of concentrated nitric acid at 105°C for 4 h and samples diluted 10 times by volume with ultrapure water (18.2 MΩ) before analysis by ICP-MS. Indium (In) was added to the nitric acid used for digestion as an internal standard to assess errors in the processes of sample treatment and analysis. The diluted samples were introduced into an ICP-MS (NexION 350D; PerkinElmer) using an SC-4 DX autosampler and Apex-HF system (Elemental Scientific Inc., Omaha, NE, United States). After raw data was obtained, plant sample weights and elemental concentrations per mass of plant material of all samples were calculated using previously described algorithms (Lahner et al., 2003).

### Linkage Mapping and Bulk Segregant Analysis

A subset of marker-validated 91 VanC RILs (Recombinant Inbred Lines) (Fitz Gerald et al., 2014) and their parental lines Col-0 and Van-0 were analyzed for leaf Zn concentration. Each line’s leaf Zn concentration and marker information were integrated for QTL mapping performed using R/qtl in R program (Broman et al., 2003; Arends et al., 2010). The contribution of each marker accounting for the variation of Zn concentration was determined using MapMaker (Lander et al., 1987).

To determine the relationship between Zn concentration and *HMA4*, we also conducted bulk segregant analysis (BSA) using an F2 population from a cross between Col-0 × Fab-2. We chose 100 F2 plants to perform BSA analysis, the phenotype of those 100 F2 plants were sorted by leaf zinc concentration. According to the leaf zinc concentration, those 100 F2 plants were classified into a high zinc (>140 μg g^-1^ DW) and a low zinc pool (<60 μg g^-1^ DW). Genomic DNA was extracted from bulked samples of each pool and analyzed using cleaved amplified polymorphic sequence (CAPS) marker (Konieczny and Ausubel, 1993). *Dra* I restriction endonuclease was used to detect the polymorphism after PCR with primers HMA4-BSA-F and HMA4-BSA-R (**Supplementary Table S1**).

### Sequencing of *HMA4* of Col-0, Van-0 and Fab-2

The genomic DNA fragments of *HMA4* in Col-0, Van-0 and Fab-2 were amplified by overlapping PCR and sequenced. The 13 pairs of primers used for overlapping PCR of *HMA4* were designed using the online tool Overlapping Primer sets^1^ and are listed in **Supplementary Table S1**. Overlapping PCR products of *HMA4* were amplified from Col-0, Van-0 and Fab-2 genomic DNA using KOD-plus-neo DNA polymerase (TOYOBO CO., LTD. Life Science Department OSAKA JAPAN). After purification by agarose gel electrophoresis these DNA fragments were sequenced from both the 5′ and 3′ ends using the PCR primers used for amplification. The sequences of these DNA fragments were assembled using the *HMA4* genomic DNA sequence from Col-0 as a reference using Lasergene software (DNASTAR)^2^. The protein sequences of HMA4 in Col-0, Van-0 and Fab-2 were predicted from their genomic DNA sequences according to the *HMA4* gene structure in the Col-0 reference.

### Transgenic Complementation

To construct the *HMA4* gene complementation vector a 10.1 kb genomic DNA fragment of *HMA4* containing ∼6.8 kbs of the gene body and ∼3.3 kbs of the promoter region were amplified from Col-0 using the HMA4-F-EcoRI and HMA4-R-BamHI primers (**Supplementary Table S1**). Amplified fragments were cloned into *pCR-XL-TOPO* vector (ThermoFisher Scientific^3^) for sequencing. *HMA4* fragments with the correct sequence from *pCR-XL-TOPO-HMA4* were reconstructed into the expression vector *pHB* using the restriction enzymes *Eco*R I and *Bam*H I. This expression vector containing the fully reconstructed *HMA4* sequence was introduced into the *A. thaliana* accession Fab-2 through *Agrobacterium tumeraciens*-mediated floral dip transformation (Clough and Bent, 1998). Positive transgenic lines were identified after screening on medium containing 50 mg/ml hygromycin B.

### Quantitative Real-Time PCR

Plants used for extracting RNA were grown for 3-week on 1x Hoagland medium containing 1% sucrose solidified with 1.2% agar. The samples were harvested in the same day. Roots or leaves from 1 plate (approximately 30 plants) for each line were pooled to generate 1 biological replicate for RNA extraction, and for each line 4 biological replicates were independently harvested. Total RNA was extracted using the TRIzol Plus RNA Purification kit (ThermoFisher Scientific)^3^. First strand cDNA synthesis was performed using the SuperScript VILO cDNA Synthesis Kit (ThermoFisher Scientific)^3^. For quantitative PCR (qPCR), 3 technical replicates of each biological replicate were analyzed on a Real-Time PCR System (ABI StepOnePlus, Applied Biosystems lco., United States) using SYBR Green PCR Master Mix (Applied Biosystems, United States) with the first strand cDNA as a template. The average of the threshold cycle (CT) values of 3 technical replicates was used to represent the CT value of one biological replicate. The primers (**Supplementary Table S1**) used for qPCR were designed using Primer Express Software version 3.0 (Applied Biosystems, United States). The amplification efficiency of the qPCR primers was confirmed to be very close to 2 (1.98) by gradually diluting the template. The relative expression of *HMA4* was calculated using 2^-ΔCT^ relative to the reference gene *Ubiquitin-conjugating enzyme 21* (At5g25760) according to previous study (Livak and Schmittgen, 2001).

### GWAS and Statistical Analysis

GWAS was performed using in a linear model and a mixed model as previously described (Chao et al., 2012). All statistical analyses used in this study were performed using R (Version 3.4.0)^4^. Phenotypic differences among *A. thaliana* accessions were compared by ANOVA and Tukey *post hoc* test using the R internal statistical functions of “aov” and “TukeyHSD”, respectively. The frequency distributions of leaf Zn concentrations and all the boxplots were plotted by the external R packages “ggplot2”.

## Results

### Natural Variation in Leaf Zn Concentration of a World-Wide Collection of *A. thaliana* Accessions

**FIGURE 1:**
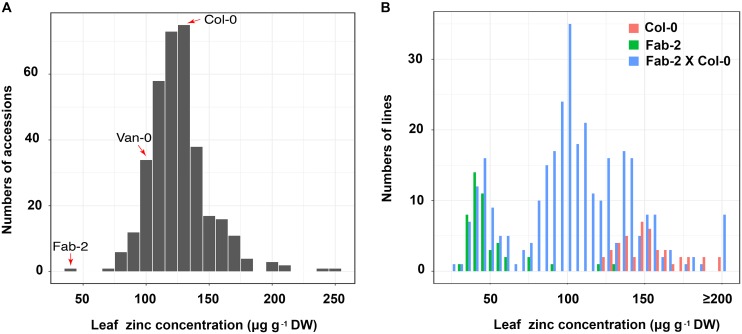
Zn concentration in a worldwide collection of *A. thaliana* accessions and F2 population of Col-0 × Fab-2 grown in a common garden. **(A)** The frequency distribution of leaf Zn content of 349 *A. thaliana* accessions grown in a controlled environment common garden. The red arrows indicate the three accessions we used in this study, *n* = 6 for each accession. **(B)** The frequency distribution of Zn content in Col-0 × Fab-2 F2 population. 316 F2 plants were used in this analysis, and *n* = 44 and 48 for Col-0 and Fab-2 respectively.

We have used ionomics coupled with genome-wide association (GWA) analysis and linkage mapping to successfully identify multiple genes that control natural variation in leaf elemental contents in *A. thaliana* (Rus et al., 2006; Baxter et al., 2008, 2010; Chao et al., 2012, 2014b; Forsberg et al., 2015). In this study, we observed that leaf Zn concentration is also highly varied, ranging from 40 to 300 μg⋅g^-1^ dry weight, among a world-wide collection of accessions of *A. thaliana* when grown in a controlled environment common garden (**Figure 1A** and **Supplementary Table S2**). We performed GWA mapping of leaf Zn concentration, but did not detect any significant association using either the linear model or the mixed model approaches. Probably, this is a result of rare alleles controlling the variation in leaf Zn concentration, which is similar to what we have observed for the GWA mapping of leaf sulfur and selenium concentrations (Chao et al., 2014a). In such a situation, linkage mapping may be more suitable than GWA mapping for identification of the genetic basis underlying this natural variation. Among the 349 accessions analyzed, Fab-2, an accession collected from Faberget, Sweden, had the lowest leaf Zn concentration of the 349 accessions analyzed (**Figure 1A**). In addition, the accession Van-0 was also observed to have ∼30% lower leaf Zn concentration than Col-0 (**Figure 1A**). As a Recombinant Inbred Lines (RIL) population derived from a cross between Van-0 and Col-0 is publically available, we chose both Fab-2 and Van-0 for further linkage mapping of the loci driving these low leaf Zn phenotypes.

### Genetic Mapping of the Locus Controlling Low Leaf Zn in Van-0 and Fab-2

**FIGURE 2:**
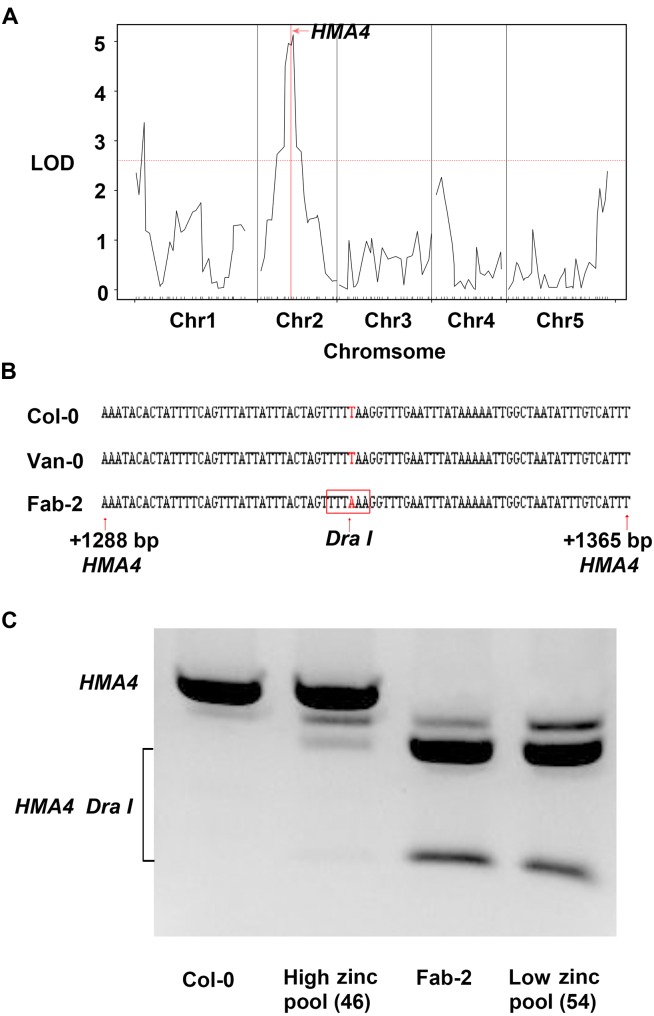
Quantitative trait loci (QTL) mapping and bulk segregant analysis (BSA) reveal *HMA4* as the best candidate gene that is responsible for the natural variation of leaf zinc concentration. **(A)** Result of QTL analysis using VanC RIL population. The red horizontal dashed line represents the threshold value of LOD, and the vertical solid red line indicates the position of *HMA4*. **(B)** The T to A substitution generated a *Dra*I restriction as a CAPS marker in Fab-2. The DNA sequence in the red box is the restriction site of *Dra*I in *HMA4* of Fab-2. **(C)** Genotyping *HMA4* in Col-0, Fab-2 and low zinc and high zinc pools from Col-0 × Fab-2 F2 indicated *HMA4* is co-segregated with zinc phenotype.

In order to identify the loci responsible for the low leaf zinc concentration in Van-0 we used 91 lines, a subset of the RIL population derived from a Col-0 × Van-0 (VanC) cross (Fitz Gerald et al., 2014), for QTL mapping. Plants were grown in a controlled environment growth room and leaf Zn concentration of 5-week-old plants measured by ICP-MS. Leaf Zn concentrations of the RILs (**Supplementary Table S3**) and their genotypic data were analyzed using the qtl R package (Broman et al., 2003). We detected two significant loci correlated with variation in leaf Zn concentration, one localized on chromosome 1 and the other on chromosome 2 (**Figure 2A**). Between the two loci, the locus on chromosome 2 contributes to 29.8% of the total variation in leaf Zn concentration in this RIL population. Interestingly, this QTL, with a likelihood of odds (LOD) value of 5.14, is co-localized with *HMA4*, a gene encoding a Zn/Cd transporter that loads Zn into the xylem for long-distance transport to the shoot (Hussain et al., 2004; Hanikenne et al., 2008; Morel et al., 2009). Though the QTL interval is as large as 2 Mb (from 7.65 Mb to 9.65 Mb on Chromosome 2) and contains 463 genes, only *HMA4* was previously reported to be involved in Zn homeostasis (**Supplementary Table S5**). We thus hypothesized that *HMA4* is the causal gene of low leaf Zn concentration in Van-0.

For mapping the causal loci controlling low leaf Zn concentration in Fab-2, we generated an F2 mapping population by crossing Fab-2 with Col-0, an accession that contains average leaf Zn concentration. A total of 316 F2 individuals, together with their parents Fab-2 and Col-0, were grown in a controlled environment growth room and their leaf Zn concentrations analyzed after grown for 5 weeks. The frequency distribution of leaf Zn concentration in all F2 individuals was plotted, and the data showed that ∼1/4 of F2 individuals had leaf Zn concentrations similar to Fab-2, and ∼3/4 of the F2 individuals were similar to Col-0 or exhibited a phenotype between Col-0 and Fab-2. This result suggested that a single recessive locus controls the low leaf Zn phenotype of Fab-2 (**Figure 1B**). To test if this locus is *HMA4*, which we identified as a strong candidate controlling low leaf Zn in Van-0, we designed a polymorphic CAPS marker on the *HMA4* intron (**Figure 2B**) that discriminated the Fab-2 and Col-0 alleles of *HMA4*, and used it to perform a bulk segregation analysis (BSA). We separately pooled 54 F2 individuals with the lowest leaf Zn concentration and 46 F2 individuals with the highest leaf Zn concentration, and genotyped these two pools for the Fab-2 and Col-0 alleles of *HMA4*. We found that the low Zn pool shares the same *HMA4* genotype as Fab-2 while the high Zn pool was a mixture of Fab-2 and Col-0 *HMA4* genotypes (**Figure 2C**). This BSA indicated that the low leaf Zn phenotype co-segregated with the homozygous Fab-2 *HMA4* allele, suggesting that the low zinc phenotype of Fab-2 is likely controlled by *HMA4* similar to Van-0.

### Genetic Complementation of Fab-2 and *hma4-2*

**FIGURE 3:**
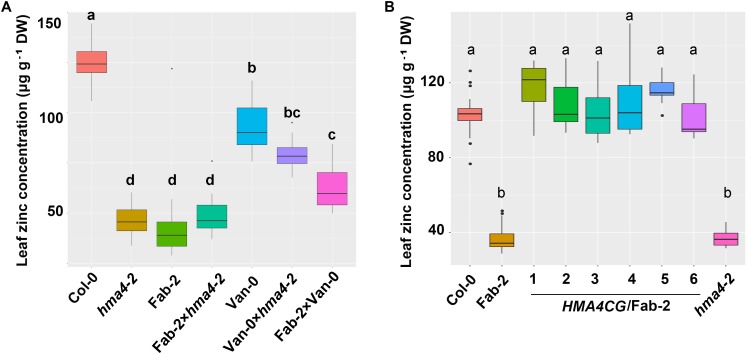
Genetic and transgenic complementation of the low leaf zinc concentration phenotype of Fab-2 and *hma4-2*. **(A)** Leaf Zn concentration in Col-0, Van-0, Fab-2, *hma4-2* and their F1 progeny. **(B)** The *HMA4* genomic fragment in Col-0 background (*HMA4CG*) can complement the low Zn phenotype of Fab-2. Significant differences were determined by one-way ANOVA following Tukey *post hoc* test. Different letters above boxplots represent significant difference at *p* < 0.05, *n* = 7–16 for each genotype.

To confirm our hypothesis that *HMA4* is the causal locus contributing to the natural variation in leaf Zn concentration, we crossed Fab-2 and Van-0 with *hma4-2* (SALK_050924), a null T-DNA insertion mutant in the Col-0 background. Analysis of leaf Zn concentration showed that *hma4-2* exhibited a similar low leaf Zn concentration as Fab-2, with 40% lower Zn concentration in the leaves compared to Col-0. The F1 progenies of a cross between *hma4-2* and Fab-2 had leaf Zn concentrations indistinguishable from Fab-2 and *hma4-2* (**Figure 3A**). This genetic data indicates that *HMA4* is responsible for the low leaf Zn phenotype of Fab-2. We also performed a genetic complementation experiment crossing Van-0 with *hma4-2* and Fab-2 separately, and observed that all of the F1 progenies showed a leaf Zn concentration between their corresponding parents (**Figure 3A**). These results support that both the low leaf Zn concentration of Fab-2 and Van-0 are driven by variation at the *HMA4* locus.

To further confirm that *HMA4* is the causal locus, we carried out transgenic complementation with Fab-2. We cloned the 10.1-kb fragment of *HMA4* genomic DNA including a 2.6-kb promoter region from Col-0 and introduced it into Fab-2. Six independent transgenic lines were identified and leaf Zn concentration measured in plants of the T2 generation. Our data showed that the Col-0 *HMA4* genomic DNA fragment is able to fully complement the low leaf Zn concentration of Fab-2 (**Figure 3B**). These results established that *HMA4* is the causal gene for the low leaf Zn concentration of Fab-2.

### Expression of *HMA4* Is Decreased in Fab-2 and Van-0

**FIGURE 4:**
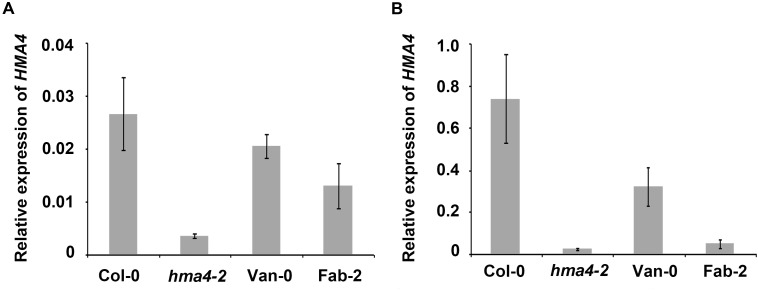
Expression level of *HMA4* in shoot **(A)** and root **(B)** of Col-0, Van-0, Fab-2 and *hma4-2*. For the analysis *UBC* (AT5G25760) was used as an internal standard. The expression of *HMA4* was calculated as 2^-ΔCT^ relative to *UBC*. Data represent means ± SE, *n* = 4.

There have been numerous examples where polymorphisms in promoter regions drive intra species specific natural variation in various traits by affecting expression levels of the causal genes, such as *ATPS1, AtHKT1* and *AtMOT1* (Rus et al., 2006; Baxter et al., 2008; Koprivova et al., 2013). To examine if *HMA4* is a similar case, we checked the expression level of *HMA4* in Fab-2 and Van-0 using qPCR. We observed that *HMA4* is primarily expressed in the root, as transcript levels in roots are 25-fold higher than those in the shoot (**Figure 4**), which is consistent with previous studies (Hussain et al., 2004; Sinclair et al., 2007). Interestingly, we found that expression of *HMA4* in Fab-2 roots is decreased by 93.5% while that in Van-0 is decreased by 56.5% compared with that in Col-0 root (**Figure 4B**). This finding suggests that polymorphisms in the promoters alter expression of *HMA4*, contributing to the low leaf Zn phenotype of Fab-2 and Van-0.

### Sequence Analysis Reveals the Causal Polymorphism of *HMA4* in Fab-2

**FIGURE 5:**
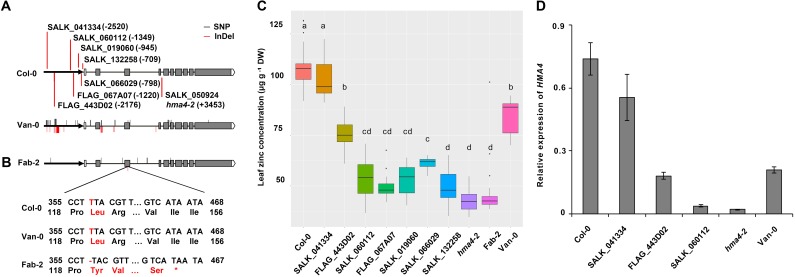
Sequence integrities of both protein and promoter of *HMA4* are essential for zinc concentration. **(A)** Insertion sites of the *HMA4* T-DNA mutants in Col-0 and the sequence variation of *HMA4* in Van-0 and Fab-2. The bold black arrows represent the promoter region, the black horizontal solid lines represent the intron and the gray boxes represent the exon, the write boxes are the 5′ UTR and the pentagons mean the 3′ UTR. The bold vertical red lines are indicate the insertion sites of T-DNA mutants. The red solid lines indicate the InDels while the black solid lines indicate the SNPs in the *HMA4* of Van-0 and Fab-2 compared with Col-0. **(B)** Coding sequence and the protein sequence of the region nearby the T deletion in the third exon of Fab-2. **(C)** Leaf Zn concentration of Fab-2, *HMA4* T-DNA mutants and Col-0. Significant differences were determined by ANOVA following Tukey *post hoc* test. Different letters above boxplots represent significant difference at *p* < 0.05, *n* = 12–24 for each genotype. **(D)** The expression of *HMA4* in different T-DNA insertion lines of *HMA4*, Col-0 and Van-0. *UBC* (AT5G25760) was used as an internal standard. The expression of *HMA4* was calculated as 2^-ΔCT^ relative to *UBC*. Data represent means ± SE, *n* = 4.

In order to examine the causal polymorphisms for the low leaf Zn concentration in Fab-2 and Van-0, we sequenced the whole *HMA4* genomic region including promoter, gene body region and 322 bp downstream of Fab-2 and Van-0. Sequencing results showed that there are 8 polymorphic sites between Fab-2 and Col-0 *HMA4* with 7 single nucleotide polymorphisms (SNPs) and 1 deletion (**Figure 5A**). Among the 7 SNPs, one is located at -2297-bp (2297-bp upstream to the start codon) and the other 6 are localized in 3 different introns (**Figure 5A** and **Supplementary Table S4**). The 1-bp deletion is in the third exon, which results in a frame shift from the 119th amino acid and a premature stop codon after the 154th residue (**Figure 5B**). This major change in protein sequence indicates that the 1-bp deletion causes loss-of-function of Fab-2 *HMA4* and is responsible for the low leaf Zn concentration of Fab-2. The large expression decrease of *HMA4* in Fab-2 root might be the result of nonsense-mediated transcripts decay.

### Sequence Analysis Suggests That the Promoter Polymorphisms Are Responsible for the Weak Function of *HMA4* in Van-0

Polymorphisms between Van-0 and Col-0 are much more extensive than between Fab-2 and Col-0. Polymorphic sites between Fab-2 and Col-0 and between Van-0 and Col-0 are less than those between Fab-2 and Van-0, indicating that the *HMA4* variation in Fab-2 and Van-0 occur independently. A total of 32 polymorphic sites were identified between Van-0 and Col-0, including 23 SNPs and 9 insertion/deletion (InDel) polymorphisms (**Figure 5A** and **Supplementary Table S4**). Among the 23 SNPs, 11 are in the promoter or 5′ untranslated region, 6 are in introns and 6 in exons. Five of the six SNPs in exons are synonymous substitutions, and only one causes an amino acid change from Col-0 Arg^805^ to Van-0 Lys^805^. While 4 InDel polymorphic sites are in the promoter region, including a 13-bp deletion and a 59-bp insertion at -2-kb site. The other 5 InDel polymorphic sites are all in introns (**Figure 5A** and **Supplementary Table S4**).

The Arg^805^ to Lys^805^ polymorphism is not in a conserved region of the HMA4 protein (Ueno et al., 2010), and accordingly this SNP is probably not responsible for the weak function of Van-0 *HMA4.* We sequenced the cDNA of *HMA4*, but did not find any splicing errors. Given most of the polymorphisms in intron are SNPs or small InDels, we speculate that these polymorphisms in introns might not affect the function of Van-0 *HMA4*. As we observed that the expression of *HMA4* in Van-0 root is reduced compared to Col-0, we propose that the polymorphisms in the promoters might be responsible for the reduced function of Van-0 *HMA4.*

### Phenotypic Analysis of T-DNA Insertion Mutants Maps the *Cis*-elements of *HMA4* and Indicates the Causal Polymorphisms of *HMA4* in Van-0

Among those polymorphisms in the promoter region, the large InDel polymorphisms ∼2-kb upstream from the start codon is a major sequence variation and thus might be the causal polymorphism for the reduced function of *HMA4* in Van-0. To test this hypothesis we obtained 7 T-DNA insertion mutants in the Col-0 background with different insertion sites within the putative promoter of *HMA4*. We grew and measured leaf Zn concentration of these mutants together with wild-type Col-0 and plants with null alleles of *HMA4*, namely *hma4-2* and Fab-2. We found that the 5 mutants (SALK_132258, SALK_066029, SALK_019060, FLAG_067A07 and SALK_060112) with T-DNA inserted in between the region of -1349-bp and -709-bp show similar or slightly higher leaf Zn concentrations compared with *HMA4* null mutants (**Figures 5A,C**). This indicates that some key cis-element(s) controlling the expression level of *HMA4* localize in the genomic region upstream to -1349-bp. However, the mutant FLAG_443D02 with a T-DNA insertion at -2176-bp exhibited an 87.5% higher leaf Zn concentration compared with the null mutants (**Figures 5A,C**), demonstrating that the region between -1349-bp and -2176-bp also contains functional *cis*-element(s) of *HMA4*. Furthermore, the mutant SALK_041334 with a T-DNA inserted at -2520-bp had no significant difference in leaf Zn concentration compared to wild-type Col-0 (**Figures 5A,C**). This result indicates that the region from -2520-bp to -2176-bp also contains cis-element(s) required for expression of *HMA4*.

To confirm the leaf Zn phenotype of the mutants, we further examined the expression of *HMA4* of some of the T-DNA insertion lines, including SALK_041334, FLAG_443D02, SALK060112 and the null mutant *hma4-2*. The qPCR results are consistent with our phenotypic observation. The expression of *HMA4* in SALK_041334 is just slightly reduced compared with Col-0, while the expression of *HMA4* decreased ∼50% in FLAG_443D02, and more than 95% in SALK_019060 and the *hma4-2* null mutant (**Figure 5D**). These results further demonstrated that a *cis*-element regulating expression of *HMA4* exists in the promoter region between -1349-bp and -2176-bp. Mapping of such a *cis*-element is consistent with our hypothesis that the large InDel polymorphisms at the -2-kb site is responsible for the low expression of *HMA4* in Van-0. However, the region between -2176-bp and -1349-bp contains many other polymorphic sites including SNP and small InDel polymorphisms which we could not exclude as contributing to variation in the function of *HMA4* in Van-0.

### Reduced *HMA4* Function Enhances Tolerance to an Environment With High Levels of Both Zn and Cd

During evolution, genetic variation could be neutral or adaptive. If *HMA4* experienced an environmental selection, the independent hypofunctional alleles in Fab-2 and Van-0 should contribute to their adaptation to certain environment. As HMA4 functions in long-distance transport of Zn and Cd (Mills et al., 2010), a hypothesis would be that impaired HMA4 might enhance tolerance to high Cd condition. However, a previous study showed that there was no observable phenotypic difference of the *hma4-2* null mutant when exposed to elevated Cd concentration in the growth medium compared to wild-type (Hussain et al., 2004). Consistently, we found that growth of Fab-2 and Van-0 as well as *hma4-2* was indistinguishable from Col-0 when plants were exposed to 10, 20, or even 40 μM Cd in the growth medium (**Supplementary Figure S1**). We also treated plants with high concentration of Zn in the growth medium. Both Fab-2 and Van-0 appeared to be more tolerant to high concentration of Zn, though there is no significant difference between Col-0 and *hma4-2* (**Supplementary Figure S2**), suggesting that factors other than *HMA4* might be involved in the elevated Zn resistance of Fab-2 and Van-0.

**FIGURE 6:**
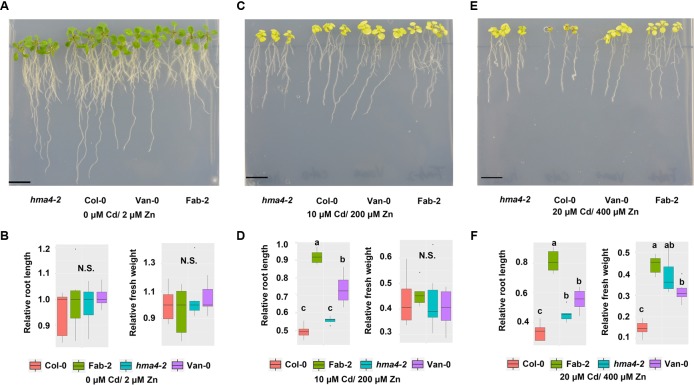
Phenotypes of *hma4-2*, Col-0, Van-0 and Fab-2 treated with both high Zn and Cd. Phenotypes in control condition (0 μM Cd/2 μM Zn) **(A,B)**, 10 μM Cd/200 μM Zn **(C,D)** and 20 μM Cd/400 μM Zn **(E,F)**. Scale bar = 15 mm, significant differences were determined by ANOVA following Tukey *post hoc* test. Different letters above boxplots represent significant difference at *p* < 0.05.

Cd and Zn often co-locate in pedosphere. Given that Zn and Cd are known to be both transported by HMA4 (Hussain et al., 2004; Wong and Cobbett, 2009; Mills et al., 2010), we wondered that whether loss-of-function of *HMA4* provided any protection from toxicity when plants are exposed to high Cd and high Zn together in the growth medium. We then treated Col-0, *hma4-2*, Van-0 and Fab-2 with different combinations of Zn and Cd in the growth medium at concentrations of 0 μM Cd/2 μM Zn, 10 μM Cd/200 μM Zn and 20 μM Cd/200 μM Zn. Our data showed that Fab-2, Van-0 and *hma4-2* are more resistant to 20 μM Cd/400 μM Zn in the growth medium, compared to Col-0, as evaluated by both relative root length and relative fresh weight (**Figure 6**).

## Discussion

Using a core collection of 349 wild collected *A. thaliana* accessions (Baxter et al., 2010; Platt et al., 2010), we observeda wide range of natural variation in leaf Zn concentration. However, we failed to identify any significant genome - wide associations for leaf Zn concentration using GWA mapping, a strategy that we have successfully used to identify *HKT1*,*MOT1, HAC1* and *HMA3* controlling natural variation in leaf concentrations of Na, Mo, As and Cd (Baxter et al., 2010; Chao et al., 2012, 2014b; Forsberg et al., 2015). GWA mapping is based on a statistical analysis of the relationship between genotype and phenotype and is not efficient in identification of rare alleles, even with large-effect (Korte and Farlow, 2013). An example of this is our study of natural variation in leaf sulfur and selenium concentrations in *A. thaliana*. Though we could not identify any genome-wide associations for either leaf sulfur or selenium concentrations using GWA mapping, we were able to identify *APR2* as the causal gene in controlling natural variation in these two traits using linkage mapping in a bi-parental F2 mapping population (Chao et al., 2014a). In this current study, our failure to detection significant associations for leaf Zn concentration is probably attributable to the same cause. We therefore mapped the causal gene by QTL analysis and BSA.

Our QTL and BSA analyses suggested that *HMA4* is the causal locus for the low leaf Zn concentration in *A. thaliana* accessions Fab-2 and Van-0. This conclusion was further confirmed by genetic and transgene complementation. Sequencing the whole genome region of *HMA4* in Fab-2 and Van-0 reveals many polymorphisms. However, the Fab-2 and Van-0 share few polymorphisms compared with Col-0, demonstrating that the mutation of *HMA4* in Fab-2 and Van-0 occur independently. There are only a few of SNPs and a 1-bp deletion polymorphism between Col-0 *HMA4* and Fab-2 *HMA4.* The 1-bp deletion localizes in the third exon results in a frame-shift of Fab-2 *HMA4*, and we propose that the Fab-2 *HMA4* is a null allele, and that this deletion is the causal polymorphism of the low leaf Zn concentration of Fab-2.

Unlike Fab-2 and *hma4-2*, Van-0 has only a mild reduction in leaf Zn concentration and is thus probably has a weak allele of *HMA4*. Sequence analysis and expression data supported this hypothesis, given *HMA4* is downregulated and numerous polymorphisms in its promoter region were observed. To further support our conclusion, we developed a strategy to map the regulatory region of *HMA4*. By using a series of T-DNA insertional lines with T-DNA insertions in the predicted promoter region, we identified two possible transcriptional regulatory regions for *HMA4*. The mapped regions overlap with the major polymorphic sites between Col-0 and Van-0, suggesting that this strategy has potential for studying gene regulation. Mapping cis-elements of a gene *in vivo* is always a challenge. This finding not only mapped the regulatory cis-elements of *HMA4* for the first time, but might be useful for mapping regulatory elements of other functional genes, as numerous T-DNA insertion mutants can be easily obtained.

In addition to *HMA4* on chromosome 4, we also identified a significant QTL on chromosome 1 controlling the variation in leaf Zn concentration of Van-0. This QTL interval is large and lacks obvious candidate genes, making it difficult to determine the causal gene without further genetic work. However, we did identify several transporters in this region, including 2 predicted members of the ZIP family of zinc transporters. Further investigations into the possible role of these candidate genes are necessary to clarify if variations in these genes also play a role in the lower leaf zinc concentration we observed in Van-0.

Natural variation is a powerful genetic resource for studying both gene function and the genetic basis of local adaptation. To date, a few adaptive genes have been characterized in plants, including *OsTT1, ACD6* and *MOT1* (Todesco et al., 2010; Poormohammad Kiani et al., 2012; Li et al., 2015). The adaptive role, if any, of the hypo functional alleles of *HMA4* we describe here, remains an interesting and open question.

Overall, this study reveals that natural variation in leaf zinc concentration within the *A. thaliana* species is governed in part by variation of *HMA4*. Interestingly, inter species specific variation in leaf Zn concentrations has also been established to be related to alteration in *HMA4* function with the Zn hyperaccumulating species *A. halleri* and *Noccaea caerulescens* having enhanced *HMA4* expression compared to non-accumulating relatives (Courbot et al., 2007; Craciun et al., 2012). This data suggests that selection acts at *HMA4* to drive variation in leaf Zn concentrations both within and between species.

## Author Contributions

D-YC and DS conceived this work and designed the experiments. Z-RC and D-YC performed most of the experiments. Z-RC, D-YC, and DS wrote the manuscript. All authors edited and commented on the manuscript.

## Conflict of Interest Statement

The authors declare that the research was conducted in the absence of any commercial or financial relationships that could be construed as a potential conflict of interest.

## Footnotes

1 http://pcrsuite.cse.ucsc.edu/Overlapping_Primers.html

2 http://www.dnastar.com

3 http://www.thermofisher.com

4 https://cran.r-project.org/

## Supplementary Material

FIGURE S1 Phenotypes of *hma4-2*, Col-0, Van-0 and Fab-2 treated with both high contents of Cd. Phenotypes in 10 μM Cd **(A–B)**, 20 μM Cd **(C–D)** and 40 μM Cd **(E–F)**. Significant differences were determined by ANOVA following Tukey *post hoc* test. Different letters above boxplots represent significant difference at *p* < 0.05.

FIGURE S2 Phenotypes of *hma4-2*, Col-0, Van-0 and Fab-2 treated with both high contents of Zn. Phenotypes in 200 μM Zn **(A–B)**, 400 μM Zn **(C–D)** and 600 μM Zn **(E–F)**. Significant differences were determined by ANOVA following Tukey *post hoc* test. Different letters above boxplots represent significant difference at *p* < 0.05.

TABLE S1 The primers used in this study.

TABLE S2 The Zn contents of 349 *A. thaliana* accessions.

TABLE S3 The Zn contents of VanC RILs.

TABLE S4 Variations of HMA4 of Fab-2 and Van-0 compared to Col-0.

TABLE S5 Candidate genes of QTL mapping using VanC RILs.
