# Supplementary material for: AtHMA4 Drives Natural Variation in Leaf Zn Concentration of Arabidopsis thaliana
Source: Front Plant Sci. 2018 Mar 1;9:270. doi: 10.3389/fpls.2018.00270 (PMC5839161; doi:10.3389/fpls.2018.00270)
Supplement: FIGURE S1 — Phenotypes of hma4-2, Col-0, Van-0 and Fab-2 treated with both high contents of Cd. Phenotypes in 10 μM Cd (A–B), 20 μM Cd (C–D) and 40 μM Cd (E–F). Significant differences were determined by ANOVA following Tukey post hoc test. Different letters above boxplots represent significant difference at p < 0.05. [file Image_1.PDF]

Supplementary Material

AtHMA4 drives natural variation in leaf Zn concentration of *Arabidopsis thaliana*

Zi-Ru Chen[1, 2], Lu Kuang[1, 3], Yi-Qun Gao[1, 2], Ya-Ling Wang[1], David E. Salt[4, *] and Dai-Yin Chao[1, *]

***Correspondence:**
Dai-Yin Chao: dychao@sibs.ac.cn

David E. Salt: david.salt@nottingham.ac.uk

Supplementary Figures

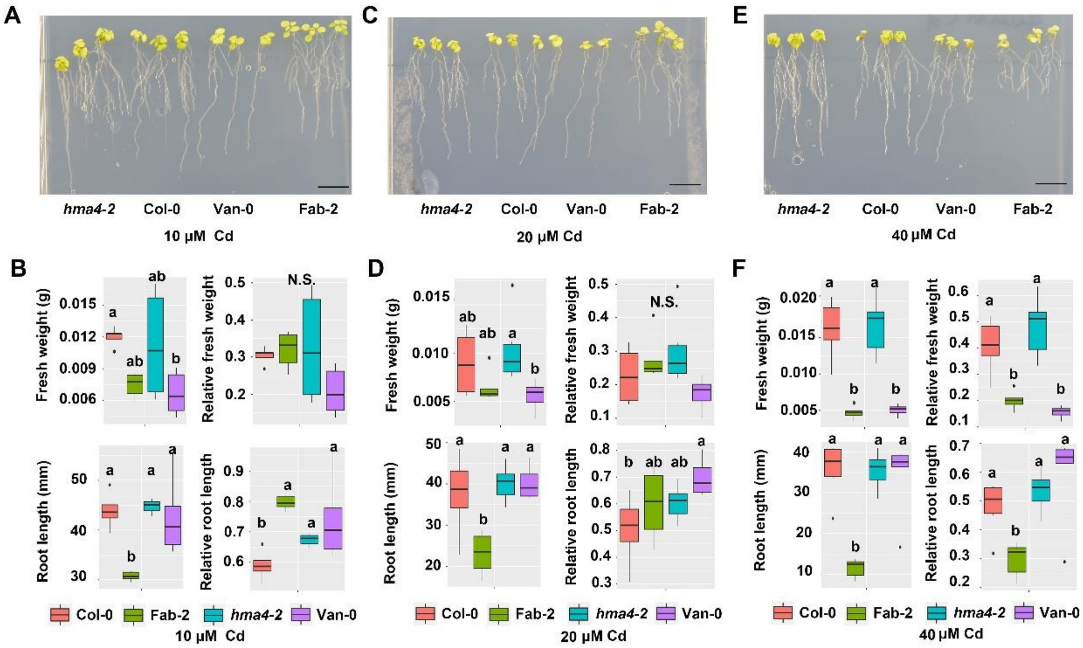

Supplementary Figure 1. Phenotypes of *hma4-2*, Col-0, Van-0 and Fab-2 treated with both high contents of Cd. Phenotypes in 10µM Cd (A-B), 20µM Cd (C-D) and 40µM Cd (E-F). Significant differences were determined by ANOVA following Tukey post hoc test. Different letters above boxplots represent significant difference at $p < 0.05$.

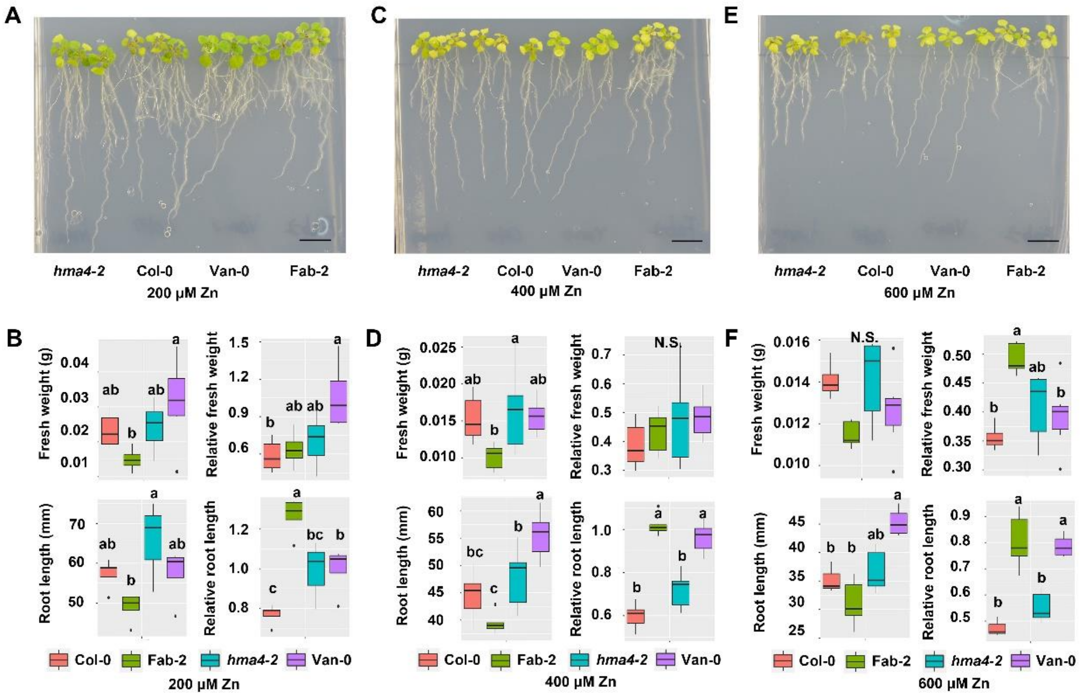

Supplementary Figure 2. Phenotypes of *hma4-2*, Col-0, Van-0 and Fab-2 treated with both high contents of Zn. Phenotypes in 200µM Zn (A-B), 400µM Zn (C-D) and 600µM Zn (E-F). Significant differences were determined by ANOVA following Tukey post hoc test. Different letters above boxplots represent significant difference at $p < 0.05$.
